# Diagnostic and Therapeutic Challenges in Pseudoangiomatous Stromal Hyperplasia (PASH) of the Breast in a 14-Year-Old Girl: A Case-Based Review

**DOI:** 10.3390/diagnostics15182395

**Published:** 2025-09-20

**Authors:** Patrycja Sosnowska-Sienkiewicz, Przemysław Mańkowski, Danuta Januszkiewicz-Lewandowska

**Affiliations:** 1 Department of Pediatric Surgery, Medical University of Warsaw, Żwirki i Wigury 63A Street, 02-091 Warsaw, Poland; 2 Department of Pediatric Surgery, Traumatology and Urology, Poznan University of Medical Sciences, ul. Fredry 10, 61-701 Poznan, Poland; mankowskip@ump.edu.pl; 3 Department of Pediatric Oncology, Hematology and Transplantology, Poznan University of Medical Sciences, ul. Fredry 10, 61-701 Poznan, Poland; danuta.januszkiewicz@ump.edu.pl

**Keywords:** adolescent, biopsy, breast neoplasms, breast surgery, diagnostic imaging

## Abstract

**Introduction:** Pseudoangiomatous stromal hyperplasia (PASH) is a benign breast lesion characterized by stromal myofibroblast proliferation forming slit-like pseudoangiomatous spaces. Although most frequently diagnosed in premenopausal women, it has also been reported in adolescent girls, where it may present as a rapidly enlarging mass that mimics fibroadenoma or phyllodes tumor. The pathogenesis is thought to be hormonally influenced, particularly by progesterone, with a possible role for estrogen. **Case Report:** We report the case of a 14-year-old girl who presented with a painless, rapidly growing mass in the left breast, first noticed approximately six months earlier. Clinical examination revealed a mobile lesion about 10 cm in diameter without skin changes, lymphadenopathy, or nipple discharge. Ultrasound and MRI demonstrated a large, well-circumscribed solid tumor (10.4 × 11.2 × 4.2 cm^3^) displacing normal breast tissue but without infiltration; both were classified as BI-RADS 4. Given the tumor size, diagnostic uncertainty, and potential risk of a non-representative core needle biopsy, a decision was made to proceed with primary radical excision. The mass was completely removed with preservation of the glandular tissue. Histopathology confirmed PASH, described macroscopically as a solid, gray–yellow, encapsulated tumor and microscopically as slit-like spaces lined by spindle cells (CD34+, CD31–). Postoperatively, the breast gradually regained symmetry with the contralateral side, and at 14 months of follow-up, no recurrence was observed. **Conclusions:** PASH, although benign, may present as a large breast tumor in adolescents and clinically mimic both benign and malignant lesions. Histological evaluation based on an adequately performed biopsy is crucial for accurate diagnosis. Complete excision with capsule preservation is recommended to minimize the risk of recurrence. In adolescents, a watchful waiting approach after surgery may be beneficial, as breast tissue often remodels and regains symmetry spontaneously, reducing the need for reconstructive procedures. This case underscores the importance of individualized diagnostic and therapeutic strategies in managing rare benign breast lesions in pediatric patients.

## 1. Introduction

Pseudoangiomatous stromal hyperplasia (PASH) of the breast is a proliferation of myofibroblasts that microscopically resembles vessel-like spaces. It is a benign lesion, although it can present as a large or tumor-like mass. The exact mechanism is not fully understood. PASH is considered a hormone-dependent lesion, particularly influenced by progesterone and estrogen, which may explain its occurrence in young women during puberty (hormonal instability), in premenopausal women, and in rare cases in men undergoing hormonal treatment [1].

The nodular form of PASH is a sporadic lesion diagnosed in the breast. It was first described by Vuitch, Erlandson, and Rosen in 1986 [1]. It most commonly occurs in middle-aged, premenopausal women, with a diagnosis frequency of approximately 6% among other benign breast lesions [2,3]. The age at diagnosis ranges from 14 to 74 years. It is exceptionally rare in adolescents, with only about 20 cases reported in this group [4].

PASH is even rarer in males and usually represents an incidental histological finding in biopsies performed for other conditions, although it may present clinically as a firm breast mass [5].

PASH usually presents as a painless lump or thickening in the breast. Although it typically does not cause pain, it may cause tenderness if the lesion is large. It is often discovered incidentally during routine breast exams or diagnostic evaluations performed for other breast changes [6].

Although rare in adolescents, it may present as a rapidly growing breast mass, which can clinically mimic a phyllodes tumor or fibroadenoma. Cases of massive PASH requiring surgical resection due to mass effect, significant breast size asymmetry, or pain have been reported in adolescent girls [6].

Diagnosis in adolescents with breast lesions includes breast ultrasound, which typically shows a well-circumscribed, hypoechoic mass. It may resemble a fibroadenoma or phyllodes tumor. Magnetic resonance imaging (MRI) is primarily employed to evaluate the extent and anatomical boundaries of the lesion. A core needle biopsy is essential to exclude malignancy, especially when inconclusive imaging or features raise concern [7]. Histopathologically, PASH is characterized by vessel-like spaces lined by spindle cells without the presence of endothelial cells (which helps differentiate it from angiosarcoma). Immunohistochemistry typically shows positive staining for fibroblast markers (CD34+) and negative staining for endothelial markers (CD31–) [3].

Management can be conservative if the lesion is small and asymptomatic. Surgical excision is recommended in cases of significant tumor growth, persistent symptoms, or diagnostic uncertainty—particularly when the lesion is large and biopsy may not provide representative tissue [1,3].

Recurrence is uncommon, especially if the lesion is completely excised. Current evidence does not suggest that PASH increases the risk of future breast malignancy [4].

## 2. Case Report

A 14-year-old girl presented to the oncology outpatient clinic after detecting a lump in her left breast during self-examination. She had noticed progressive enlargement of the breast about six months earlier, but after reading online that some degree of breast asymmetry is normal, she did not report the issue to her parents, especially since the lesion was painless.

She had no significant past medical history, was not taking any medications, had never been pregnant, and was not using hormonal contraception.

On clinical breast examination, a mobile, centrally located mass measuring approximately 10 cm was palpable, without skin changes, palpable lymphadenopathy, or pathological nipple discharge.

Ultrasound (US) revealed an extensive, solid lesion with smooth contours measuring 10.4 × 11.2 × 4.2 cm^3^, with visible vascular flow, no signs of necrosis or cystic areas, and a homogeneous internal structure. The lesion did not infiltrate the surrounding tissues.

The normal fatty–glandular breast tissue was displaced peripherally (laterally and inferiorly) by the described mass. Imaging was classified as BI-RADS category 4.

The right breast showed no abnormalities. No abnormalities were found on chest X-ray or abdominal ultrasound.

**Figure 1:**
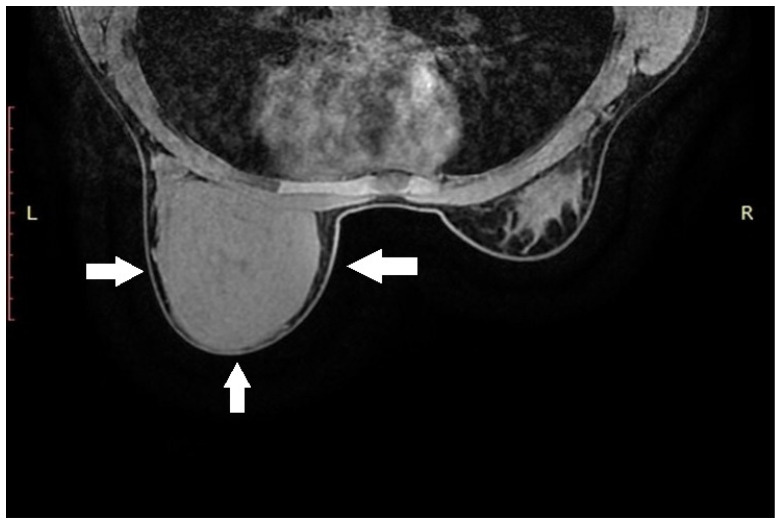
Magnetic resonance imaging of the left breast. Axial T1-weighted contrast-enhanced sequence reveals a large, homogeneous tumorous lesion, indicated by white arrows.

Due to the size of the lesion, diagnostic evaluation was extended to include magnetic resonance imaging (MRI). MRI confirmed the previous findings, showing a well-circumscribed lesion without diffusion restriction. No additional focal pathological changes were detected. No pathological lymph nodes were visualized in either axilla (levels I, II, or III). MRI was classified as BI-RADS category 4 for the left breast and category 1 for the right breast (Figure 1).

The overall clinical picture in the described patient suggested a lesion most consistent with a phyllodes tumor or fibroadenoma. In the absence of features suspicious for malignancy, a decision was made to proceed with primary radical excision of the tumor.

Through a ~4 cm incision beneath the left breast, the mass was removed using a combination of blunt and sharp dissection, with complete preservation of the glandular tissue. The lesion did not infiltrate the surrounding tissues.

The skin was closed with a continuous non-absorbable suture, and a compression dressing was applied. The patient was discharged two days later without complications.

**Figure 2:**
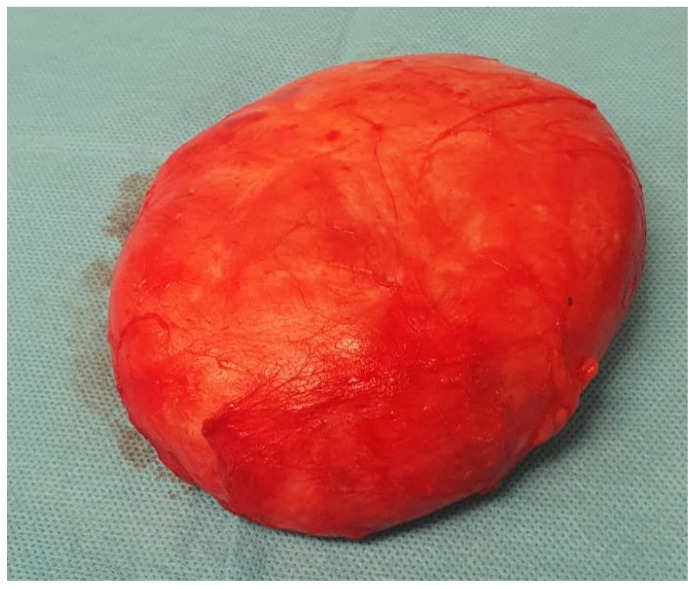
Resected surgical specimen.

**Figure 3:**
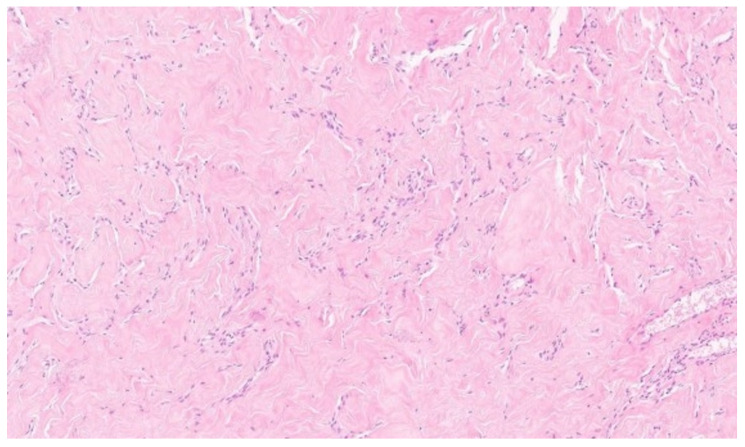
Histological images of the PASH. Multiple interconnected slit-like pseudoangiomatous spaces, lined by spindle-shaped cells and separated by thick, hyalinized collagen bundles. H and E, 10×.

The histopathological examination confirmed the diagnosis of pseudoangiomatous stromal hyperplasia (PASH). The specimen was described as a solid, light gray-to-yellow tumor measuring 11 × 10 × 4.8 cm^3^, with the capsule preserved and intact (Figure 2 and Figure 3).

The patient was subsequently followed up in outpatient care, and after approximately 12 weeks, the operated breast had regained a size comparable to the contralateral side, without the need for plastic surgery to remove excess soft tissue resulting from the prior expansive tumor growth.

**Figure 4:**
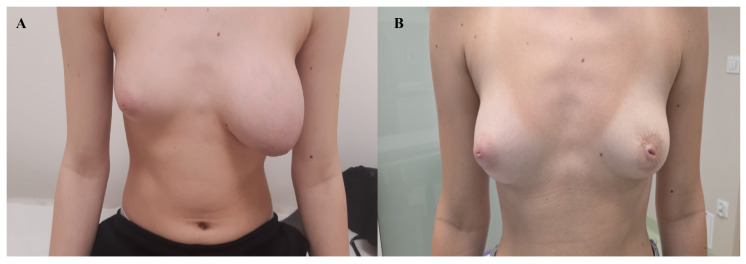
The patient at the time of diagnosis (**A**) and one year after surgical treatment (**B**).

The patient’s current physical examination and medical history show no abnormalities. In a follow-up ultrasound performed seven months postoperatively, both breasts presented a normal appearance (Figure 4).

## 3. Discussion

While PASH is commonly found incidentally in association with other benign or malignant pathologies, its presentation as a dominant, nodular mass is rare-particularly in adolescents [6,8]. Based on a study by Pellini et al., the available data include 27 adolescent patients [4].

Our 14-year-old patient is particularly noteworthy not only because of her young age and the massive size of the tumor, but also because breast conservation was achieved without the need for reconstructive surgery. This case further demonstrates that, even in massive lesions, complete excision with preservation of the native glandular tissue may be sufficient. The follow-up observation of spontaneous remodeling and restoration of breast symmetry underscores the potential of the adolescent breast to regain normal appearance without additional corrective procedures.

In women, PASH is primarily associated with hormonal factors, particularly progesterone. The lesion most often occurs in premenopausal women and in patients using hormonal contraception or hormone replacement therapy (exposure to synthetic hormones may influence the proliferation of stromal myofibroblasts, which frequently express progesterone receptors). Pregnancy and lactation may also play a role [1,6,9]. PASH has occasionally been reported in patients with diabetes or lupus [5,10].

The patient treated by our team had none of the above risk factors. The only possible explanation for the occurrence of the tumor in her case appears to be the dynamic hormonal changes of puberty, which are also considered a potential risk factor for PASH development [4,6,11].

Clinically, PASH usually presents as a firm, painless, and mobile solitary mass, without associated nipple or skin changes. Rarely, it may be diffuse or multinodular. PASH typically ranges between 0.6 cm and 12 cm, with most cases being small to medium. It may present along a broad clinicopathologic spectrum, from incidental focal findings to symptomatic palpable breast masses [12]. In young patients, it typically manifests as a rapidly growing palpable lesion [6,11]. This was the case in our 14-year-old patient: a rapidly enlarging (reported history of approximately six months), painless mass, without other associated symptoms, measuring 10.4 × 11.2 × 4.2 cm^3^.

Mammography of PASH lesions reveals a well-circumscribed, dense, homogeneous mass without calcifications. However, the application of mammography in adolescents is limited due to the fibrous nature of breast tissue, which may hinder lesion detection or lead to misinterpretation of normal development as suspicious findings [4]. Sosnowska-Sienkiewicz et al. suggest that ultrasonography is an excellent, non-burdensome initial diagnostic method, with MRI reserved for inconclusive cases [7]. Pellini et al. state that neither ultrasound nor MRI are sufficiently specific to establish a definitive diagnosis. Cytology rarely provides diagnostic confirmation; therefore, histological examination remains necessary [4]. Given the often challenging interpretation of imaging in young breasts, we agree with this position, emphasizing the importance of excisional biopsy, particularly in adolescent patients with suspected phyllodes tumor, which may have both benign and malignant components [7,13].

In our patient, although ultrasound and MRI demonstrated a well-circumscribed lesion with features suggestive of a benign process, the imaging results were not definitive and could not reliably distinguish between fibroadenoma, phyllodes tumor, and PASH. The massive size of the lesion (over 10 cm) and its BI-RADS 4 classification further limited the diagnostic certainty. A core needle biopsy is the standard of care in the evaluation of breast masses. In our case, however, the decision to proceed directly to surgical excision resulted from a combination of factors. The patient presented with a very large, centrally located mass (over 11 cm in diameter), and the imaging findings were suggestive but not diagnostic, raising suspicion of a phyllodes tumor. Considering the known heterogeneity of phyllodes tumors-with possible coexistence of benign and malignant histologic areas- a limited core biopsy might not have captured regions of higher-grade pathology. This is supported by reports in the literature indicating that sampling errors are more likely in large lesions with variable internal architecture [7]. Furthermore, due to size-related symptoms and cosmetic concerns, the mass was to be excised entirely regardless of the biopsy result.

Importantly, the decision was made by a multidisciplinary team consisting of pediatric oncologists, breast surgeons, and radiologists, fully considering the patient’s comfort, psychological well-being, and diagnostic efficiency. Our approach in this case was individualized and is not intended to suggest deviation from established standards of care in general practice.

This concern is significant in suspected phyllodes tumors, which may harbor areas of different histological grades—benign, borderline, or malignant—within the same lesion [7]. Therefore, limited sampling could have underestimated the lesion’s biological potential. For these reasons, and given the clinical suspicion of a phyllodes tumor, complete surgical excision was selected as the most reliable diagnostic and therapeutic approach. Similar diagnostic dilemmas have been described in the literature, where lesions initially interpreted as fibroadenomas, phyllodes tumors, or hamartomas were ultimately confirmed histopathologically as PASH [4,7,13].

Currently, there are no formal guidelines for the management of PASH. Available treatment options include conservative management, local excision, or wide surgical excision with mastectomy in cases of mass effect [4]. According to Bouche et al., symptomatic and palpable lesions require surgical excision, whereas in smaller lesions, discontinuation of hormonal contraception with reassessment after six months may be considered. However, validation of this approach in larger cohorts is necessary [1].

Some reports suggest the use of tamoxifen (off-label) as a non-surgical treatment for symptomatic PASH, given its presumed hormonal etiology. Pruthi et al. and Seltzer et al. reported rapid symptom relief and reduction in breast volume following tamoxifen therapy, although efficacy waned after three months in one case [14,15]. However, all reported cases involved adult women. To date, no evidence of tamoxifen use in adolescent patients has been found in the literature. Moreover, ethical concerns limit its applicability in this age group, as tamoxifen may interfere with growth, pubertal development, and future fertility. Therefore, while tamoxifen may represent a temporary option in selected adult patients, its role in adolescents remains highly questionable and should not be recommended outside of clinical research settings.

On gross examination, PASH typically appears as a well-circumscribed and encapsulated breast lesion, occasionally presenting in a diffuse form. The cut surface is smooth, firm, or rubbery, glistening, and varies in color from gray to tan-pink, yellow, or white [4]. In our patient, the lesion was described as a solid, light gray–yellow mass.

Microscopically, the hallmark of PASH is the presence of slit-like spaces resembling vascular channels, lined by spindle cells, without endothelial markers (CD31–), but with positive CD34 expression, which was also confirmed in our case [4,16].

Sporadic cases of atypical PASH have been described in the literature. These are characterized by cytological alterations of myofibroblasts, manifesting as myofibroblastic sarcoma arising from PASH [17].

Although PASH is generally benign, it tends to recur if incompletely excised. Therefore, surgical resection with careful attention to removing the lesion together with its capsule is required, while striving for breast conservation. Regardless of the benign nature of the lesion and its favorable prognosis, long-term follow-up is recommended, as recurrences have been reported in 7–22% of cases [18].

Our patient underwent radical excision with preservation of the tumor capsule, and no recurrence was observed over 14 months of follow-up.

A watch-and-wait strategy after surgery proved effective—the operated breast regained volume similar to the contralateral side, which is particularly important in adolescent patients, in whom preserving breast development and avoiding reconstructive surgery, when possible, should remain a priority. In cases where reconstruction is necessary, exceptionally careful patient selection is required [19]. As with other surgical procedures, credentials for performing cosmetic surgery should be based on education, training, experience, and demonstrated competence [20].

Our experience with the presented patient highlights the importance of an individualized diagnostic and therapeutic approach to rare benign breast lesions in pediatric patients (Supplementary Materials: Table S1).

## 4. Conclusions

PASH, although benign, may present as a large breast tumor in adolescents and clinically mimic other benign or malignant lesions. Histological evaluation based on an adequately performed biopsy is crucial for accurate diagnosis. A complete excision with capsule preservation is recommended to reduce the risk of recurrence. In adolescent patients, adopting a watchful waiting approach after surgery may be particularly beneficial, as demonstrated in our case: despite the massive size of the tumor, breast conservation was achieved, and subsequent follow-up revealed spontaneous tissue remodeling with restoration of symmetry, thereby eliminating the need for reconstructive procedures. This case underscores the importance of individualized diagnostic and therapeutic strategies in managing rare benign breast lesions in pediatric patients.

## Data Availability

Due to restrictions, the data supporting this publication are available upon reasonable request from the corresponding author.

## Supplementary Materials

The following supporting information can be downloaded at https://www.mdpi.com/article/10.3390/diagnostics15182395/s1, Table S1. Key facts about pseudoangiomatous stromal hyperplasia (PASH).
