# Supplementary material for: Diagnostic and Therapeutic Challenges in Pseudoangiomatous Stromal Hyperplasia (PASH) of the Breast in a 14-Year-Old Girl: A Case-Based Review
Source: Diagnostics (Basel). 2025 Sep 20;15(18):2395. doi: 10.3390/diagnostics15182395 (PMC12469236; doi:10.3390/diagnostics15182395)
Supplement: Supplementary file 1 [file diagnostics-15-02395-s001.zip › diagnostics-3867488-supplementary.pdf]

Supplementary Table S1. Key facts about pseudoangiomatous stromal hyperplasia (PASH).

Category	Summary
Epidemiology	Rare benign breast lesion, most common in premenopausal women; incidence ~6% among benign breast biopsies. Reported age range: 14–74 years. Very rare in adolescents (≈20–30 cases described). Extremely rare in males, usually incidental finding (often in gynecomastia).
Pathogenesis/Risk factors	Hormone-dependent lesion. Associated with progesterone and estrogen exposure (oral contraceptives, HRT, pregnancy, lactation). Occasional reports in patients with diabetes or lupus.
Clinical presentation	Usually a firm, painless, mobile breast mass; often solitary. Size typically 0.6–12 cm (occasionally very large). May present as diffuse or multinodular enlargement. In adolescents: commonly a rapidly enlarging mass mimicking fibroadenoma or phyllodes tumor.
Imaging features	Ultrasound: well-circumscribed, hypoechoic, homogeneous mass; may resemble fibroadenoma.<br>Mammography: dense, circumscribed, homogeneous lesion without calcifications (limited role in adolescents).<br>MRI: used to evaluate lesion extent; non-specific, cannot reliably distinguish PASH from fibroadenoma/phyllodes tumor.
Histopathology	Slit-like, pseudoangiomatous spaces lined by spindle-shaped myofibroblasts (not endothelial cells).<br>IHC profile: CD34+, PR/ER+, vimentin+; CD31–, D2-40–.

Management	Small, asymptomatic lesions: conservative management with follow-up.<br>Symptomatic/large/rapidly growing/uncertain diagnosis: surgical excision (local or wide).<br>Mastectomy: rarely indicated (massive or diffuse cases).<br>Recurrence: 7–22% if incompletely excised.<br>Experimental: Tamoxifen reported in adults, but not validated in adolescents (ethical concerns).
Prognosis	Benign lesion, does not appear to increase breast cancer risk. Long-term follow-up recommended, especially in young patients.
